# Remnant Gastric Necrosis after Laparoscopic Distal Gastrectomy Managed with Double-Elemental Diet Tube and Laparoscopic Gastrojejunostomy: A Case Report

**DOI:** 10.70352/scrj.cr.26-0041

**Published:** 2026-04-18

**Authors:** Kazuki Sameshima, Takaaki Arigami, Daisuke Matsushita, Masataka Shimonosono, Hiroshi Okumura, Keishi Okubo, Masahiro Noda, Ken Sasaki, Kenji Baba, Takao Ohtsuka

**Affiliations:** 1 Department of Digestive Surgery, Kagoshima University Graduate School of Medical and Dental Sciences, Kagoshima, Kagoshima, Japan; 2 Department of Surgery, Southern Region Hospital, Makurazaki, Kagoshima, Japan

**Keywords:** remnant gastric necrosis, laparoscopic distal gastrectomy, double-elemental diet tube, laparoscopic gastrojejunostomy

## Abstract

**INTRODUCTION:**

Although the stomach has a robust intramural microvascular network and is generally considered resistant to ischemia, remnant gastric necrosis after gastrectomy is very rare and potentially fatal. However, diagnosis can be challenging, and optimal management strategies are still not well established.

**CASE PRESENTATION:**

A 78-year-old man with hypertension, arteriosclerosis, chronic renal dysfunction, chronic obstructive pulmonary disease, internal carotid artery stenosis on antiplatelet therapy, long-term heavy smoking, and daily alcohol intake presented with anemia. Esophagogastroduodenoscopy (EGD) revealed a type 3 tumor in the lower stomach. Histopathological examination of biopsied specimens revealed moderately differentiated adenocarcinoma with poorly differentiated components. Preoperative imaging revealed severe vascular calcification of the abdominal aorta and splenic artery. The patient underwent laparoscopic distal gastrectomy with D1+ lymphadenectomy and Billroth I reconstruction (delta-shaped anastomosis). Postoperatively, he developed fever and elevated C-reactive protein levels without clinical or radiological evidence of peritonitis or shock. Contrast-enhanced CT and EGD revealed circumferential mucosal necrosis in the distal remnant stomach. A double-elemental diet (W-ED) tube was placed for intragastric decompression and enteral feeding. A minor anastomotic leak resolved with conservative management. However, a severe anastomotic stricture required an elective laparoscopic gastrojejunostomy (Billroth II with Braun anastomosis). The patient recovered and remained disease-free with stable oral intake for 4 years.

**CONCLUSIONS:**

Conservative management using a W-ED tube, followed by elective gastrojejunostomy for stricture, may be a viable treatment option for remnant gastric necrosis after distal gastrectomy in patients with high surgical risk when peritonitis is absent and systemic status is stable.

## Abbreviations

CRP
: C-reactive protein
DG
: distal gastrectomy
EBD
: endoscopic balloon dilation
EGD
: esophagogastroduodenoscopy
ICG
: indocyanine green
LDG
: laparoscopic distal gastrectomy
PGA
: posterior gastric artery
W-ED
: double-elemental diet

## INTRODUCTION

DG with lymphadenectomy is the standard surgical procedure for gastric cancer. However, minimally invasive approaches, such as laparoscopic and robotic techniques, are now widely adopted.^1)^ Common reconstruction methods after DG include Billroth I, Billroth II, and Roux-en-Y.^1)^ The stomach receives blood supply from 5 major arteries and several branches, including the PGA and left subdiaphragmatic arteries. After DG, the short gastric artery becomes the primary source of blood flow to the remnant stomach and is supplemented by these branches.^2)^ Despite the extensive microvascular network of the stomach, postoperative complications, such as bleeding, anastomotic leakage, and stricture, are relatively common, whereas remnant stomach necrosis is exceedingly rare. The mortality rate can reach 30%, even after complete resection of the residual stomach.^3–8)^

Herein, we report a case of remnant gastric necrosis following LDG with Billroth I reconstruction for locally advanced gastric cancer that was successfully managed by laparoscopic gastrojejunostomy after intragastric decompression using a W-ED tube.

## CASE PRESENTATION

**Fig. 1:**
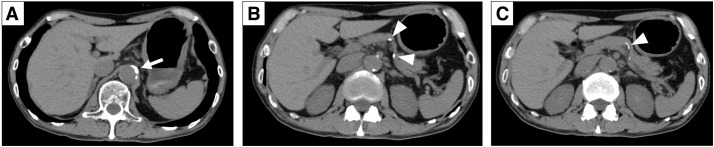
Preoperative contrast-enhanced CT images. (**A**–**C**) Severe arterial calcification of the abdominal aorta (arrow) and splenic artery (arrowheads).

A 78-year-old man with hypertension, arteriosclerosis, chronic renal dysfunction, chronic obstructive pulmonary disease, and internal carotid artery stenosis on antiplatelet therapy (cilostazol) was referred for anemia (hemoglobin, 11.5 g/dL). He had a 40-year history of heavy smoking (60 cigarettes/day, which he stopped 18 years prior) and daily alcohol consumption of 360 mL for 58 years. EGD revealed a type 3 tumor on the anterior wall of the lower third of the stomach, and histopathological examination of biopsied specimens confirmed moderately differentiated adenocarcinoma with poorly differentiated components (tub2 > por). Endoscopic ultrasonography showed third-layer interruption and fourth-layer invasion. CT revealed severe arterial calcification of the abdominal aorta and splenic artery without lymph nodes or distant metastases (**Fig. 1A**–**1C**). The patient was clinically diagnosed with stage I gastric cancer (cT2N0M0).

**Fig. 2:**
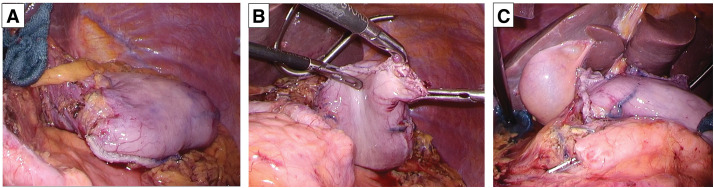
Intraoperative findings in LDG. (**A**) Residual stomach size. (**B**) Pre-reconstruction appearance. (**C**) Post-reconstruction appearance. LDG, laparoscopic distal gastrectomy

After preoperative heparin replacement for cilostazol, which had been discontinued 3 days before surgery, LDG with D1+ lymphadenectomy and Billroth I reconstruction were performed using a delta-shaped anastomosis technique. The short gastric and left subdiaphragmatic arteries were preserved, and the PGA was transected. The PGA was intentionally transected to increase the mobility of the remnant stomach, as tension at the delta-shaped anastomosis was anticipated if the vessel were preserved. The gastrectomy was completed at the midline of the stomach, leaving approximately half of the upper stomach intact (**Fig. 2A**). During the reconstruction, the remnant stump appeared slightly pale with minor oozing of serous fluid (**Fig. 2B** and **2C**). A closed drain was placed between the remnant stomach and liver. Operation time was 337 min, with blood loss of 20 mL. The final pathological diagnosis was pT2N2M0, corresponding to pStage IIB according to the Japanese Classification of Gastric Carcinoma. Prophylactic anticoagulation with unfractionated heparin was administered postoperatively.

**Fig. 3:**
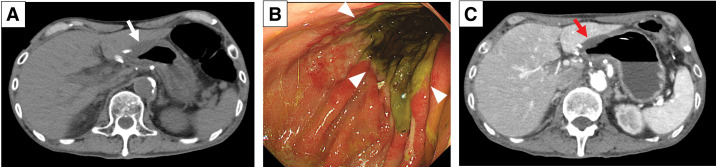
Postoperative CT images and endoscopic findings. (**A**) Marked edematous changes (white arrow) of the remnant stomach wall (POD 3). (**B**) Circumferential mucosal necrosis (arrowheads) of the distal remnant stomach (POD 8). (**C**) Poor enhancement (red arrow) of the distal remnant stomach (POD 14).

**Fig. 4:**
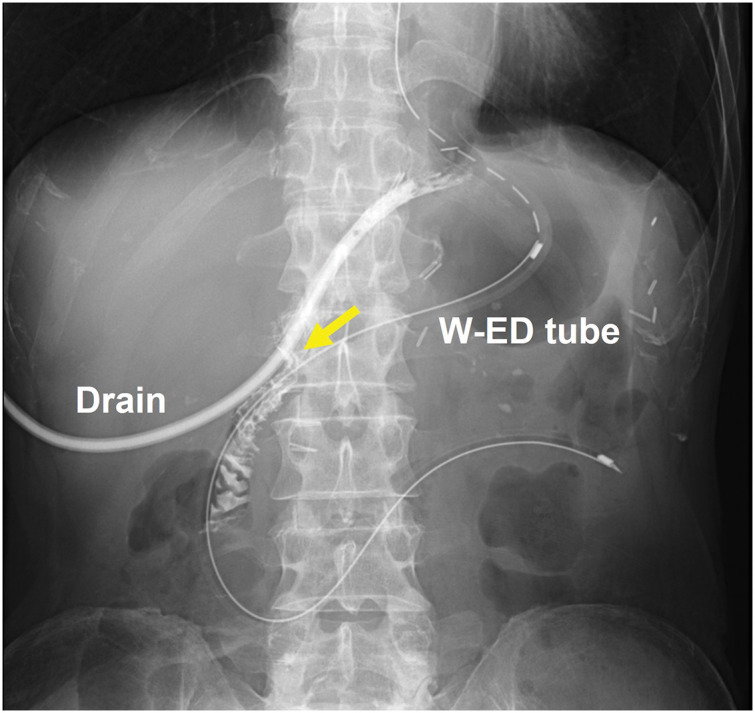
Upper gastrointestinal series via a drain. Minor anastomotic leakage (yellow arrow) was identified (POD 21). W-ED, double-elemental diet

On POD 2, the patient developed a fever of >38°C. On POD 3, the CRP level increased to 19.4 mg/dL. CT showed marked edema of the remnant stomach wall (**Fig. 3A**). There were no signs of generalized peritonitis or shock, and the drain output remained clear. EGD performed on POD 8 revealed circumferential mucosal discoloration with loss of mucosal integrity confined to the distal remnant stomach, without evidence of deep ulceration, perforation, full-thickness defects, or exposure of the muscularis propria (**Fig. 3B**). A W-ED tube (W-ED Tube; Cardinal Health, Tokyo, Japan) was used for intragastric decompression and enteral feeding. Contrast-enhanced CT on POD 14 showed poor enhancement of the distal remnant stomach (**Fig. 3C**), although clinical symptoms were mild and CRP was not elevated (1.3 mg/dL). On POD 21, an upper gastrointestinal series via a drain revealed a minor anastomotic leakage (**Fig. 4**). The closed drain was replaced with an open-ended drain to irrigate the abscess. After the initial surgery, oral intake was initiated for the first time on POD 33 once the leakage had completely resolved. Cilostazol was resumed after the initiation of oral intake.

**Fig. 5:**
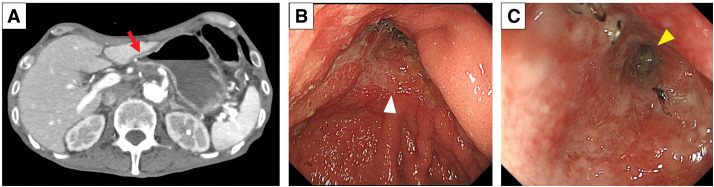
Postoperative CT images and endoscopic findings. (**A**) Improved enhancement (red arrow) of the distal remnant stomach (POD 60). (**B**) Improved mucosa (white arrowhead) of the distal remnant stomach (POD 60). (**C**) Severe anastomotic stenosis (yellow arrowhead) (POD 60).

**Fig. 6:**
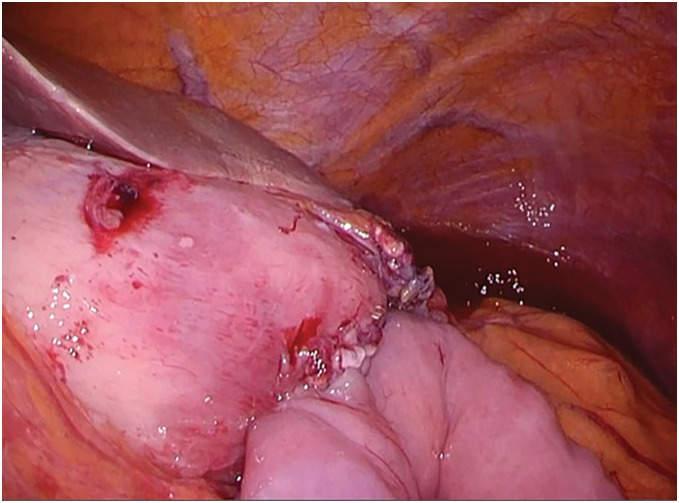
Intraoperative findings in gastrojejunostomy.

**Fig. 7:**
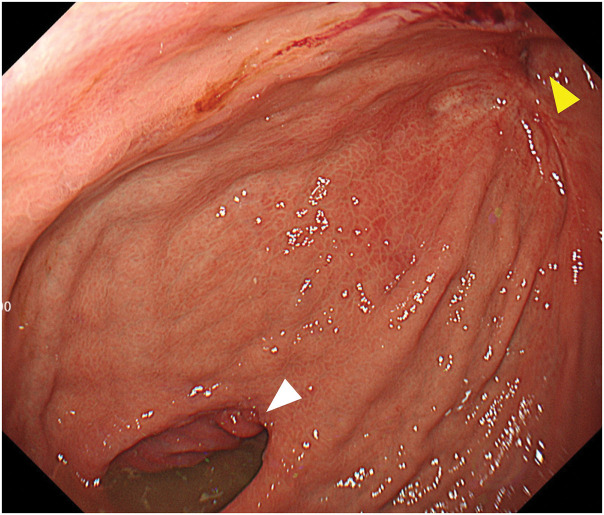
Endoscopic findings at 1 year postoperatively. Endoscopic evaluation demonstrates satisfactory patency of the gastrojejunostomy (white arrowhead) and a residual stricture at the previous Billroth I anastomotic site (yellow arrowhead).

On POD 60, contrast-enhanced CT and EGD demonstrated improved enhancement of the distal remnant stomach and severe anastomotic stenosis (**Fig. 5A**–**5C**). Despite dietary adjustments and continued W-ED tube supplementation, the patient’s oral intake remained insufficient because only liquid meals could pass through the progressively narrowing anastomosis. Serum albumin increased from 2.6 to 3.1 g/dL after initiation of W-ED tube supplementation. Therefore, a laparoscopic gastrojejunostomy was performed on POD 68. Necrosis or ischemia was not observed intraoperatively. A Billroth II gastrojejunostomy combined with a Braun anastomosis was performed using a linear stapler, with the enterotomy closed using a full-thickness running barbed suture (**Fig. 6**). Operating time was 157 min, with minimal blood loss. Following the reoperation (laparoscopic gastrojejunostomy on POD 68), oral intake was restarted on POD 4. Recovery was uneventful, and the patient was discharged on POD 7. Follow-up EGD performed at 1 year postoperatively revealed satisfactory patency of the gastrojejunostomy, although a residual stricture remained at the previous site of the Billroth I anastomosis (**Fig. 7**). The patient remained disease-free and free of dietary difficulties for 4 years postoperatively.

## DISCUSSION

Remnant gastric necrosis after DG is extremely rare despite the rich vascular supply and efforts to preserve blood flow during surgery, and the associated mortality rate remains high.^3–8)^ We present a case of remnant gastric necrosis after LDG for gastric cancer, which was successfully managed with conservative treatment using a W-ED tube, followed by elective gastrojejunostomy for stricture. To the best of our knowledge, this appears to be one of the few reported cases of remnant gastric necrosis after LDG for gastric cancer managed conservatively during the acute phase.

Known risk factors for remnant gastric necrosis include smoking, diabetes, hypertension, hyperlipidemia, and arteriosclerosis.^9)^ Our patient had multiple comorbidities, including hypertension, arteriosclerosis, chronic renal dysfunction, chronic obstructive pulmonary disease, and internal carotid artery stenosis, as well as a history of smoking and alcohol consumption. Significant alcohol consumption and smoking are strongly associated with vascular calcification and ischemic events.^10,11)^ To further clarify the underlying mechanism of remnant gastric mucosal necrosis in this patient, several pathophysiological factors likely contributed in a multifactorial manner. First, although no macroscopic vascular anomaly was identified intraoperatively, the PGA was transected. The transection of this artery may have reduced collateral perfusion to the remnant stomach after DG. Second, although the short gastric arteries and the left subdiaphragmatic artery were preserved, ischemic injury can still occur despite their preservation, as described in previous reports.^4,5)^ This may be attributable to diffuse microvascular dysfunction or diminished perfusion reserve in patients with severe arteriosclerosis. In our case, marked calcification of the abdominal aorta and splenic artery on preoperative CT suggested systemic macrovascular disease, which could have limited compensatory increases in blood flow during postoperative inflammatory stress. Third, impaired venous drainage may also have contributed. Local venous congestion resulting from postoperative edema, increased intraluminal pressure, or microvascular thrombosis can compromise mucosal perfusion even when arterial inflow appears relatively preserved. Taken together, the ischemic event in our patient was likely the result of a combination of impaired arterial supply, reduced collateral flow, and potential venous congestion rather than a single isolated factor. Furthermore, remnant gastric necrosis is extremely rare, and no reliable data comparing its frequency among Billroth-I, Billroth-II, or Roux-en-Y reconstruction, or between open and minimally invasive surgery, have been reported. Importantly, previous reports suggest that the development of remnant gastric necrosis is driven primarily by impaired gastric perfusion—such as reduced arterial inflow, inadequate collateral circulation, or venous congestion—rather than by the reconstruction method itself.^4,8)^ Additionally, the gastrectomy was completed at approximately the midline of the stomach in this case. Hosogi et al. demonstrated that setting the transection line according to fixed anatomical landmarks—such as the midpoint from the avascular zone defined by the terminal branch of the short gastric artery and the first branch of the left gastroepiploic artery to the confluence of the right and left gastroepiploic arteries—provides oncological safety while maintaining adequate blood supply to the remnant stomach.^12)^ Nonetheless, in our patient, several vascular factors—including severe arteriosclerosis with marked vascular calcification—likely diminished the perfusion reserve, even at a commonly used transection level. Based on this experience, in patients with compromised vascular anatomy or an increased risk of ischemia, careful selection of the transection line along established anatomical perfusion zones and choosing a reconstruction method that minimizes tension and preserves collateral flow may help reduce the risk of postoperative ischemic complications.

Contrast-enhanced CT is useful in evaluating postoperative complications, such as perforation and abscess, but has limited sensitivity for early ischemia, particularly at the arteriolar level.^13,14)^ In contrast, EGD allows direct visualization of mucosal ischemia and early necrotic changes, making it more reliable during the early postoperative period.^15,16)^ In this case, EGD was pivotal for diagnosis and management. Recently, ICG fluorescence imaging, which is increasingly being used in minimally invasive surgery, has gained attention for its ability to assess organ perfusion and may help prevent ischemic complications by identifying perfusion deficits intraoperatively.^17,18)^ If ICG had been applied during the index operation, earlier recognition of perfusion compromise might have prompted intraoperative conversion to a different reconstruction or more extensive resection, potentially averting necrosis. However, establishing clear criteria for the use of ICG fluorescence imaging is clinically challenging. Although intraoperative discoloration and preoperative vascular risk factors may suggest its potential usefulness, the optimal indications have not yet been standardized. Accordingly, further prospective studies are needed to clarify evidence-based criteria for ICG application in this setting.

Published case reports indicate that salvage total gastrectomy is frequently performed, but carries high mortality in patients with remnant gastric necrosis after gastrectomy.^3–8)^ Park et al. reviewed 24 patients with remnant gastric necrosis after subtotal gastrectomy and reported mortality rates of 30.8%, 80%, and 66.7% in 13 patients who underwent total gastrectomy, 5 patients who underwent jejunostomy, and 6 patients who received conservative management, respectively.^4)^ Regarding treatment strategies, most reported cases required emergency total remnant gastrectomy, whereas a smaller number were successfully managed conservatively when necrosis was limited to the mucosa and the patients remained hemodynamically stable. Unfortunately, no standardized therapeutic strategy has been established to improve prognosis, making careful monitoring of the general condition, laboratory findings, and CT or EGD findings clinically essential. To clarify our treatment strategy, our decision-making process was based on the following clinical criteria. Conservative management was continued when the patient showed no signs of generalized peritonitis, maintained stable vital signs, demonstrated improving CRP levels, and had no radiological or endoscopic evidence suggestive of transmural necrosis. Gakuhara et al. also reported a case of remnant gastric necrosis after proximal gastrectomy that was successfully managed conservatively without surgery under similar clinical conditions.^6)^ Given the patient’s multiple comorbidities and high surgical risk, conservative therapy was selected as the preferred initial approach. Conversely, conversion to surgery was considered in the event of worsening abdominal symptoms, development of diffuse peritonitis, hemodynamic instability, rising inflammatory markers, or imaging/endoscopic findings indicating progression to deep ischemic injury or uncontrolled anastomotic leakage. Most patients who ultimately required total remnant gastrectomy in previous reports exhibited at least one of these unfavorable conditions.^4)^ Furthermore, the improvement in blood flow to the remnant stomach observed during the clinical course may be explained by several pathophysiological mechanisms. The stomach possesses an extensive collateral microvascular network, and the development or recruitment of collateral channels over time may partially restore perfusion when the main arterial inflow is compromised.^2)^ Furthermore, the resolution of postoperative inflammatory edema may have led to the recovery of mucosal microcirculation. Previous reports of gastric remnant ischemia have noted that improved contrast enhancement on follow-up CT can reflect restoration of microvascular perfusion once inflammation subsides.^8)^ Another plausible mechanism is improvement in venous drainage. Venous congestion has been implicated as a contributing factor in gastric ischemia, and its resolution—through reduction of intragastric pressure or attenuation of tissue edema—may enhance mucosal blood flow.^19)^

According to the Japanese Gastric Cancer Treatment Guidelines, adjuvant S-1 therapy is generally recommended for pathological stage II gastric cancer.^1)^ Although earlier total gastrectomy could theoretically have enabled earlier initiation of S-1, several clinical studies have demonstrated that total gastrectomy is associated with poorer postoperative nutritional recovery and reduced tolerance to adjuvant chemotherapy.^20)^ Furthermore, recent comparative studies have further shown that patients who undergo total gastrectomy experience significantly higher chemotherapy-related toxicity and poorer tolerance than those with partial gastric preservation.^20)^ Although drawing definitive conclusions regarding the oncological implications of our treatment strategy is challenging, the patient has remained recurrence-free for 4 years postoperatively, suggesting that our approach did not compromise oncological outcomes.

Early enteral nutrition helps attenuate postoperative hypermetabolism, preserves intestinal barrier function, reduces infection, accelerates recovery, and reduces complications and mortality in patients with malnourished gastrointestinal cancer.^21)^ The W-ED tube is advantageous because it provides simultaneous intragastric decompression and enteral nutritional functions, which are not reliably achieved by conventional feeding tubes.^6,22–24)^ In our institution, we generally favor early placement of a W-ED tube in patients with suspected postoperative complications, such as anastomotic leakage, gastric dysfunction, and impaired gastric emptying. However, we do not follow a rigid protocol; instead, the timing is individualized based on the patient’s clinical condition, inflammatory response, and endoscopic or radiologic findings. In this case, although the patient developed fever and elevated CRP levels on POD 2–3, he exhibited no signs of generalized peritonitis or hemodynamic instability, and the drainage output remained clear. Therefore, immediate W-ED placement was deferred. The tube was inserted on POD 8, when EGD revealed circumferential mucosal necrosis, clearly indicating the need for both intragastric decompression and enteral nutritional support. This simultaneous decompression may help reduce intragastric pressure and prevent further progression of ischemia in compromised gastric tissue. In addition, continuous enteral nutrition delivered through the W-ED tube supports maintenance of mucosal integrity and overall systemic recovery during the acute phase. Previous reports, including that by Gakuhara et al., have demonstrated successful conservative management of remnant gastric necrosis under similar conditions using a W-ED tube,^6)^ suggesting that this device may play an important role in stabilizing high-risk patients who are not immediate candidates for reoperation. In our case, the W-ED tube contributed to stabilization and facilitated the elective timing for minimally invasive gastrojejunostomy. Moreover, the choice of treatment for the anastomotic stenosis also required careful consideration. Endoscopic balloon dilation (EBD) is commonly used for anastomotic strictures. However, in this patient, EBD was not selected because the delta-shaped anastomosis had previously developed a minor anastomotic leak and we were concerned that balloon dilation could re-perforate the fragile anastomotic site.

## CONCLUSIONS

We report a rare case of remnant gastric necrosis after LDG with Billroth I reconstruction that was successfully managed with intragastric decompression and enteral feeding via a W-ED tube during the acute phase, followed by elective laparoscopic gastrojejunostomy for severe anastomotic stricture. When peritonitis is absent and the patient’s general condition is stable, conservative management with W-ED tube support may be a viable treatment option for patients at high surgical risk.

## DECLARATIONS

### Availability of data and materials

The datasets generated in this study are available from the corresponding author upon reasonable request.
